# Point-of-Care Ultrasound for Intubation Confirmation of COVID-19 Patients

**DOI:** 10.5811/westjem.2020.7.48657

**Published:** 2020-08-17

**Authors:** Michael Gottlieb, Stephen Alerhand, Brit Long

**Affiliations:** * Rush University Medical Center, Department of Emergency Medicine, Chicago, Illinois; † Rutgers New Jersey Medical School, Department of Emergency Medicine, Newark, New Jersey; ‡ Brooke Army Medical Center, Department of Emergency Medicine, San Antonio, Texas

## Abstract

The novel coronavirus disease of 2019 (COVID-19) is associated with significant morbidity and mortality, as well as large numbers of patients requiring endotracheal intubation. While much of the literature has focused on the intubation technique, there is scant discussion of intubation confirmation. Herein, we discuss the limitations of traditional confirmatory approaches, summarize the literature supporting a role for point-of-care ultrasound in this application, and propose an algorithm for intubation confirmation among COVID-19 patients.

## BACKGROUND

Novel coronavirus disease of 2019 (COVID-19) was first identified in Wuhan, China, beginning in December 2019.^1^,^2^ Since then, the virus has spread rapidly, infecting over 13.3 million people worldwide and resulting in nearly 580,000 deaths.^2^ Hypoxemic respiratory failure requiring intubation may occur in up to 19% of all COVID-19 hospitalized patients and 70% of those admitted to the intensive care unit.^3^–^5^

COVID-19 is rapidly transmissible and, while the most common means of transmission is droplet, airborne transmission may also occur during aerosol-generating procedures such as intubation and subsequent bag-valve ventilation.^6^ While much of the transmission conversation has revolved around intubation itself,^7^ the discussion of risk associated with post-intubation endotracheal tube (ETT) confirmation is more limited. This commentary will highlight the limitations associated with current intubation confirmation techniques in light of COVID-19 and propose an alternate approach using point-of-care ultrasound (POCUS).

## LIMITATIONS OF TRADITIONAL CONFIRMATORY METHODS

Traditional methods of intubation confirmation (eg, auscultation for bilateral breath sounds, condensation in the ETT) are insufficiently accurate in isolation.^8^,^9^ Visualization of ETT passage may be limited by difficult laryngoscopic views and the use of personal protective equipment (PPE). Auscultation can also be challenging in a loud room and may not be possible with some forms of PPE. Moreover, in light of the surface stability of severe acute respiratory syndrome coronavirus 2 (SARS-CoV-2), auscultation with a stethoscope increases the potential risk for transmission via fomite exposure, while also requiring clinicians to be much closer to the patient which can increase their risk of infection.^10^,^11^

Other devices, such as end-tidal carbon dioxide (CO_2_) detectors and colorimetric capnometry, require at least five breaths for confirmation. This can lead to gastric distension and an increased risk of aspiration if the ETT is incorrectly placed in the esophagus, as well as increased risk of particle aerosolization to providers from the positive pressure ventilations.^8^,^12^ Additionally, capnography may be less reliable in patients where there is a paucity of CO_2_ produced (eg, cardiac arrest, pulmonary embolism), with studies suggesting that quantitative capnography may be only 60–65% sensitive during cardiac arrest.^13^,^14^

When assessing for mainstem (ie, endobronchial) intubation, auscultation is equally problematic, with studies demonstrating that auscultation alone may misidentify mainstem intubation in 35–60% of patients.^15^–^17^ While radiographs are typically the gold standard for assessing ETT depth, they can be significantly delayed due to the PPE necessary to perform this task and limited departmental resources, which may lead to significant lung barotrauma for unrecognized mainstem intubations in this population with limited oxygen reserve.

## ROLE OF POINT-OF-CARE ULTRASOUND FOR INTUBATION CONFIRMATION

POCUS has been increasingly recognized as a valuable tool for intubation confirmation. One approach for this is the transtracheal technique, wherein a clinician places the transducer across the patient’s neck post-intubation to visualize the ETT within the trachea or esophagus. This can be facilitated by gently twisting the ETT to create a motion artifact.^18^,^19^ A recent systematic review and meta-analysis found that transtracheal ultrasound was 99% sensitive and 97% specific for confirming ETT location among adult patients.^20^ A similar review among pediatric patients found that POCUS was 92–100% sensitive and 100% specific.^21^ Studies have also demonstrated that the accuracy remains consistent regardless of ETT size or transducer type.^22^,^23^ Additionally, the learning curve for identifying ETT placement with transtracheal POCUS has been suggested to be relatively short.^24^ Importantly, this modality offers the unique benefit that it does not require positive pressure ventilation, thereby minimizing additional exposure to staff.

Other studies have suggested using indirect signs, such as bilateral lung sliding or diaphragmatic elevation for intubation confirmation with a high degree of accuracy.^25^ Two studies demonstrated that the combination of lung sliding with transtracheal POCUS further increased the diagnostic accuracy over either in isolation.^26^,^27^

## ROLE OF POINT-OF-CARE ULTRASOUND FOR DETECTING MAINSTEM INTUBATION

Mainstem intubation can be detected through the following three sonographic assessments: lung sliding; diaphragmatic excursion; or the presence of lung pulse. In a mainstem intubation there is no air flow through the contralateral lung, resulting in the absence of the lung sliding (ie, motion artifact visualized between the visceral and parietal layers of the pleura) on that side. Studies of both cadaveric models and emergency department patients have demonstrated that unilateral right lung sliding was 69–92% sensitive and 55.6–100% specific for detecting right mainstem intubation.^28^,^29^ When compared with auscultation, this technique has outperformed auscultation in both adult and pediatric patients.^30^,^31^

Sonographic assessment of hemidiaphragmatic movement can also be used as a surrogate for ventilation of that lung. When a lung is ventilated by air, the diaphragm will move inferiorly, allowing for direct visualization of lung expansion. Studies have found that this technique is 91–100% sensitive and 50–100% specific, with near-perfect inter-rater reliability.^32^,^33^

Finally, lung pulse is the visualization of the rhythmic movement of the visceral pleura against the stationary parietal pleura resulting from cardiac pulsations through an airless and motionless left lung due to right mainstem intubation.^34^,^35^ This technique was found to be 93% sensitive and 100% specific for detecting right mainstem intubation.^34^ The lung pulse may be particularly valuable for differentiating a mainstem intubation from a pneumothorax, as both would demonstrate unilateral absence of lung sliding.

## PROPOSED ALGORITHM

We propose a rapid POCUS algorithm for confirming intubation in COVID-19 patients (Figure). First, transtracheal POCUS can be used to identify endotracheal vs esophageal intubation. If there is concern with regard to location, secondary findings (eg, lung sliding) can be used. After confirming the endotracheal location, bilateral lung sliding or diaphragmatic excursion should be used to identify whether a mainstem intubation has occurred. If there is ambiguity regarding this, lung pulse can be used to differentiate unilateral lung sliding from a pneumothorax vs a mainstem intubation. If a mainstem intubation is suggested, the clinician should slowly withdraw the ETT while visualizing the contralateral lung for the re-appearance of lung sliding. This algorithm has not been prospectively validated and future studies should assess the accuracy and safety of this approach.

## CONCLUSION

Post-intubation ETT confirmation of COVID-19 patients presents a significant risk of exposure to providers and may be more limited by PPE. We propose the integration of POCUS into the intubation confirmation pathway and present a novel algorithm. Future studies should assess the impact of this on provider safety and the diagnostic accuracy of the protocol compared with current methods.

## Figures and Tables

**Figure:**
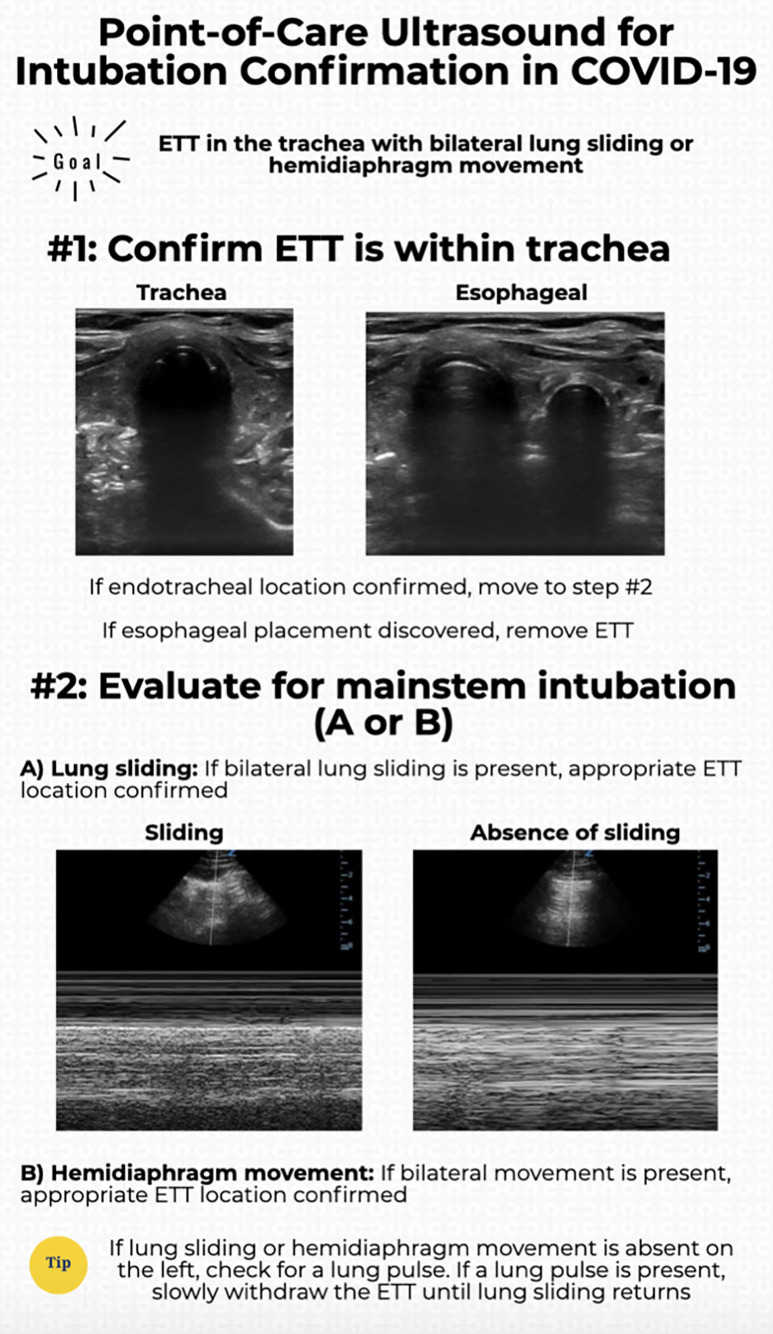
POCUS algorithm for confirming intubation in COVID-19 patients.

## References

[1] Huang C, Wang Y, Li X (2020). Clinical features of patients infected with 2019 novel coronavirus in Wuhan, China. Lancet.

[2] World Health Organization Situation Report 178.

[3] Guan WJ, Ni ZY, Hu Y (2020). Clinical characteristics of coronavirus disease 2019 in China. N Engl J Med.

[4] Wu Z, McGoogan JM (2020). [Ahead of Print]. Characteristics of and important lessons from the coronavirus disease 2019 (COVID-19) outbreak in China: summary of a report of 72314 cases from the Chinese Center for Disease Control and Prevention. JAMA.

[5] Arentz M, Yim E, Klaff L (2020). Characteristics and outcomes of 21 critically ill patients with COVID-19 in Washington state. JAMA.

[6] Centers for Disease Control and Prevention Interim Infection Prevention and Control Recommendations for Patients with Suspected or Confirmed Coronavirus Disease 2019 (COVID-19) in Healthcare Settings.

[7] Canelli R, Connor CW, Gonzalez M (2020). Barrier enclosure during endotracheal intubation. N Engl J Med.

[8] Takeda T, Tanigawa K, Tanaka H (2003). The assessment of three methods to verify tracheal tube placement in the emergency setting. Resuscitation.

[9] Kelly JJ, Eynon C, Kaplan JL (1998). Use of tube condensation as an indicator of endotracheal tube placement. Ann Emerg Med.

[10] van Doremalen N, Bushmaker T, Morris DH (2020). Aerosol and surface stability of SARS-CoV-2 as compared with SARS-CoV-1. N Engl J Med.

[11] Buonsenso D, Pata D, Chiaretti A (2020). COVID-19 outbreak: less stethoscope, more ultrasound. Lancet Respir Med.

[12] MacLeod BA, Heller MB, Gerard J (1991). Verification of endotracheal tube placement with colorimetric end-tidal CO2 detection. Ann Emerg Med.

[13] Tanigawa K, Takeda T, Goto E (2000). Accuracy and reliability of the self-inflating bulb to verify tracheal intubation in out-of-hospital cardiac arrest patients. Anesthesiology.

[14] Tanigawa K, Takeda T, Goto E (2001). The efficacy of esophageal detector devices in verifying tracheal tube placement: a randomized cross-over study of out-of-hospital cardiac arrest patients. Anesth Analg.

[15] Brunel W, Coleman DL, Schwartz DE (1989). Assessment of routine chest roentgenograms and the physical examination to confirm endotracheal tube position. Chest.

[16] Sitzwohl C, Langheinrich A, Schober A (2010). Endobronchial intubation detected by insertion depth of endotracheal tube, bilateral auscultation, or observation of chest movements: randomised trial. BMJ.

[17] Geisser W, Maybauer DM, Wolff H (2009). Radiological validation of tracheal tube insertion depth in out-of-hospital and in-hospital emergency patients. Anaesthesia.

[18] Gottlieb M, Holladay D, Burns KM (2019). Ultrasound for airway management: an evidence-based review for the emergency clinician. Am J Emerg Med.

[19] Gottlieb M, Burns K, Holladay D (2020). Impact of endotracheal tube twisting on the diagnostic accuracy of ultrasound for intubation confirmation. Am J Emerg Med.

[20] Gottlieb M, Holladay D, Peksa GD (2018). Ultrasonography for the confirmation of endotracheal tube intubation: a systematic review and meta-analysis. Ann Emerg Med.

[21] Lin MJ, Gurley K, Hoffmann B (2016). Bedside ultrasound for tracheal tube verification in pediatric emergency department and ICU patients: a systematic review. Pediatr Crit Care Med.

[22] Gottlieb M, Holladay D, Nakitende D (2019). Variation in the accuracy of ultrasound for the detection of intubation by endotracheal tube size. Am J Emerg Med.

[23] Gottlieb M, Holladay D, Burns K (2019). Accuracy of ultrasound for endotracheal intubation between different transducer types. Am J Emerg Med.

[24] Chenkin J, McCartney CJ, Jelic T (2015). Defining the learning curve of point-of-care ultrasound for confirming endotracheal tube placement by emergency physicians. Crit Ultrasound J.

[25] Chou EH, Dickman E, Tsou PY (2015). Ultrasonography for confirmation of endotracheal tube placement: a systematic review and meta-analysis. Resuscitation.

[26] Park SC, Ryu JH, Yeom SR (2009). Confirmation of endotracheal intubation by combined ultrasonographic methods in the emergency department. Emerg Med Australas.

[27] Sağlam C, Unlüer EE, Karagöz A (2013). Confirmation of endotracheal tube position during resuscitation by bedside ultrasonography. Am J Emerg Med.

[28] Weaver B, Lyon M, Blaivas M (2006). Confirmation of endotracheal tube placement after intubation using the ultrasound sliding lung sign. Acad Emerg Med.

[29] Sim SS, Lien WC, Chou HC (2012). Ultrasonographic lung sliding sign in confirming proper endotracheal intubation during emergency intubation. Resuscitation.

[30] Ramsingh D, Frank E, Haughton R (2016). Auscultation versus point-of-care ultrasound to determine endotracheal versus bronchial intubation: a diagnostic accuracy study. Anesthesiology.

[31] Sooragonda SG, Arora S, Jain D (2020). Lung sliding sign to detect endobronchial intubation in children: An observational feasibility trial. Eur J Anaesthesiol.

[32] Hsieh KS, Lee CL, Lin CC (2004). Secondary confirmation of endotracheal tube position by ultrasound image. Crit Care Med.

[33] Kerrey BT, Geis GL, Quinn AM (2009). A prospective comparison of diaphragmatic ultrasound and chest radiography to determine endotracheal tube position in a pediatric emergency department. Pediatrics.

[34] Lichtenstein DA, Lascols N, Prin S (2003). The “lung pulse”: an early ultrasound sign of complete atelectasis. Intensive Care Med.

[35] Alerhand S, Tsung JT (2020). Unmasking the lung pulse for detection of endobronchial intubation. J Ultrasound Med.

